# Supplementary material for: Novel LOX Variants in Five Families with Aortic/Arterial Aneurysm and Dissection with Variable Connective Tissue Findings
Source: Int J Mol Sci. 2021 Jul 1;22(13):7111. doi: 10.3390/ijms22137111 (PMC8269155; doi:10.3390/ijms22137111)
Supplement: Supplementary file 1 [file ijms-22-07111-s001.zip › ijms-1269133-supplementary.pdf]

Table S1: Clinical features overview LOX patients

	Family 1 p.(Leu18Profs*111)							Family 2 p.(Arg118Glyfs*119)			Family 3 p.(Gly149*)				Family 4 p.(Met298Arg)	Family 5 p.(Leu306Pro)
**Individual**	II:2	II:3	III:4	III:6	III:10	IV:1	IV:7	III:1	II:1	I:1	II:4	II:5	III:1	II:1	II:1	III:1
**Gender**	M	M	M	M	M	M	F	M	M	M	M	M	F	M	F	M
**Age (years)**	60	50	43	45	43	25	16	40	53	66	56	53	28	59	50	21
**Arterial anomalies**	Bental surgery age 60 after dissection, died 73 due to myocardial infarction	Sudden death (age 50)	Type A dissection (53mm)	Aortic dissection (age 45-84mm) and Bentall surgery	Aortic sinus diameter (43mm - age 43)	Normal diameter	Normal diameter	Type A dissection at age of 19 (underwent composite graft surgery)	Discrete mitral and aortic valve insufficiency		Borderline aortic sinus (41mm)	Mild valve regurgitation			Left carotid dissection (age 46)	Dilatation of aorta sinus and ascendens (age 6)
															Coronary artery dissection (age 44)	Elective replacement of the ascending aorta (age 14)
**Connective tissue anomalies**								Splenic rupture	Inguinal hernia surgery	Varicose veins	Inguinal hernia surgery (age 3)	Tall stature	Joint hyper-mobility	Bilateral inguinal hernia	Increased skin elasticity	Tall stature
								Spontaneous pneumo-thorax		Cataract	Rupture of supraspinatus ligament (age 41)	Narrow palate	Arachno-dactyly	Tall stature	Beighton score of 4/9	
								Varicose veins		Ischemic heart disease	Tibialis posterior neuralgia (age 42)	Pes planus	Pes planus	Joint hyper-mobility	Enlarged armspan	
										Contra-lateral and incar-cerated hernia		Inguinal hernia surgery (age 40)	Shoulder dislocations and ankle distortions		Recurrent joint dislocation	

Table S2: **ACMG classification and pathogenicity predictions of *LOX* variants**

	Gnomad	CADD	REVEL	MetaLR	PolyPhen2	Mutation-Taster	ACMG classification
**p.(Leu18Profs*111)**	Absent	-	-	-	-	Disease causing	**Pathogenic** (PVS1; PM2; PS3; PP4)
**p.(Arg118Glyfs*119)**	Absent	-	-	-	-	Disease causing	**Pathogenic** (PVS1, PS3, PM2)
**p.(Gly149*)**	Absent	34	-	-	-	Disease causing	**Pathogenic** (PVS1, PS3, PM2)
**p.(Met298Arg)**	Absent	32	0,736	0,313	Probably damaging	Disease causing	**Pathogenic** (PP3; PM1; PM2; PS1; PS3)
**p.(Leu306Pro)**	Absent	31	0,811	0,358	Probably damaging	Disease causing	**VUS** (PM1; PM2; PP3)

Table S3 part 1: Clinical features overview LOX patients literature (Guo et al., Lee et al., Cirnu et al., Renner et al.)

	Guo et al TAA-602 p.(Ser280Arg)				Guo et al TAA-111 p.(Ser348Arg)	Guo et al TAA-271 p.(Gly202*)	Guo et al TAA-703 p.(Thr248Ile)	Guo et al TAA-92291 p.(Trp42*)						Guo et al TAA-9544 p.(Gln267Pro)		
**Individual**	III:1	II:5	II:4	II:3	III:1	III:4	II:2	IV:2	II:3	II:4	III:1	II:1	III:2	III:6	II:1	III:3
**Gender**	F	M	M	F	M	M	F	M	F	M	M	M	M	M	M	M
**Age (years)**	37	62	60	54	41	36	40	11	54	/	59	70	/	16	51	37
**Arterial anomalies**	Fusiform aortic root (38mm)/ ascending aortic aneurysm (41mm)	Aortic root (52mm)/ ascending aortic aneurysm (42mm)	Type A aortic dissection (age 60)	Aortic root aneurysm (39mm)	Valve and ascending surgery (age 41); Root surgery (age 51)	Elective valve	Ascending aortic aneurysm (52mm)	Aortic root/ ascending aortic aneurysm	Thoracic aortic aneurysm and repair	Thoracic aortic aneurysm and repair	Thoracic aortic aneurysm and repair	Died after thoracic aortic surgery	Thoracic aortic aneurysm	Thoracic aortic aneurysm (Bentall surgery)	Bentall surgery	Aortic root enlargement
	Mild miltral regurgitation	Left ventricular hyper-trophy (LVH)		Ectatic ascending aorta	Ascending aortic aneurysm (125mm)	Aortic root aneurysm (42mm)	Transverse aortic arch aneurysm							BAV		
		Biscupid aortic valve (BAV)			Aortic root aneurysm	Ascending aortic aneurysm (fusiform)	BAV (age 40)									
		Three-vessel coronary artery disease (CAD)			Valve and ascending surgery (age 41); Root surgery (age 51)	Elective valve	Ascending aortic aneurysm (52mm)									
	Fusiform aortic root (38mm)/ ascending aortic aneurysm (41mm)	Aortic root (52mm)/ ascending aortic aneurysm (42mm)	Type A aortic dissection (age 60)	Aortic root aneurysm (39mm)												
**Connective tissue anomalies**								Dolicho-stenomelia						High arched palate		Dural ectasia
								Scoliosis						Joint laxity		Pectus deformity
								Pectus excavatum						Skin striae		Joint hyper-mobility
																Skin striae

Table S3 part 2: Clinical features overview LOX patients literature (Guo et al., Lee et al., Cirnu et al., Renner et al.)

	Guo et al TAA-9544 p.(Gln267Pro)		Lee et al p.(Met289Arg)					Cirnu et al p.(Cys291Ser)				Renner et al p.(Trp42*)	Renner et al p.(Tyr332Cys)
**Individual**	IV:1	I:1	III:1	II:1	II:3	III:3	III:4	II-1	II-2	II-3	I-2	Subject 188	Subject 13
**Gender**	M	M	M	F	F	M	M	M	M	F	F	/	M
**Age (years)**	14	54	35	61	73	46	41	53	36	37	74	23	48
**Arterial anomalies**	Aortic root enlargement	TAA surgery	Ascending aortic aneurysm (105mm - age 19)	TAA surgery (age 52)	TAA surgery	Aortic arch dissection (age 41)	Infrarenal abdominal aneurysm	Ascending aortic aneurysm and dissection (age 29)	Aortic aneurysm and rupture (sudden death)	Cerebral hemorrhage	Bowel infarction and stroke	Aortic aneurysm and dissection (type A)	Aortic aneurysm (ascending aortic replacement)
					Arterial tortuosity	Hepatic arterial aneurysm		Infrarenal abdominal aneurysm					
								Aortic arch aneurysm (age 46)					
								Aneurysms of A. iliaca communis, A. femoralis, A poplitea, A tibialis anterior					
								Coronary aneurysm					
**Connective tissue anomalies**	Dural ectasia	Dural ectasia	Pectus excavatum		Pectus excavatum	Abdominal hernia (age 43)		Mild scoliosis					Translucent skin
	Pectus deformity	Pectus deformity	Tall stature		Tall stature	Myopia		Tall stature					Easy bruising
	Joint hypermobility	Joint hypermobility	High arched palate		High arched palate			High arched palate					Hernia
	Skin striae	Skin striae	Skin striae		Varicose veins			Ascending aortic aneurysm and dissection (age 29)					Recurrent injuries of ankle ligaments
	Dural ectasia	Dural ectasia	Venous varicosities		Skin striae								

Table S4: Genes included in TAAD gene panel

Gene	Reference transcript (Ensembl)	Alternative exon	Reference transcript for alternative exon (Ensembl)
ABL1	ENST00000372348		
ACTA2	ENST00000458208		
ARIH1	ENST00000379887		
BGN	ENST00000331595		
COL3A1	ENST00000304636		
EFEMP2/FBLN4	ENST00000307998		
ELN	ENST00000358929		
EMILIN1	ENST00000380320		
FBN1	ENST00000316623		
FBN2	ENST00000262464		
FLNA	ENST00000369850		
FOXE3[a]	ENST00000335071		
HCN4	ENST00000261917		
LMOD1	ENST00000367288		
LOX	ENST00000231004		
LTBP3	ENST00000301873		
MAT2A	ENST00000306434		
MFAP5	ENST00000359478		
MYH11	ENST00000452625	Exon 42B	ENST00000396324
MYLK	ENST00000360304		
NOTCH1	ENST00000277541		
PLOD1	ENST00000196061	Exon 2A	ENST00000449038
PMEPA1/TMEPAI	ENST00000341744		
PRKG1[b]	ENST00000401604		
SKI	ENST00000378536		
SLC2A10	ENST00000359271		
SMAD2	ENST00000402690		
SMAD3	ENST00000327367	Exon 1A	ENST00000439724
SMAD4	ENST00000342988		
SMAD6	ENST00000288840		
TGFB2	ENST00000366929		
TGFB3	ENST00000238682		
TGFBR1	ENST00000374994		
TGFBR2	ENST00000359013		

[a]Only Forkhead domein
[b]Only exon 3
